# Professional Quality of Life and Psychological Impact on Frontline Healthcare Worker during the Fourth Wave of COVID-19

**DOI:** 10.1155/2024/2865063

**Published:** 2024-01-02

**Authors:** Hanif Ullah, Safia Arbab, Chang-Qing Liu, Sher Alam Khan, Sohail Shahzad, Ka Li

**Affiliations:** ^1^West China Hospital, West China School of Nursing, Sichuan University, Chengdu, China; ^2^Lanzhou Institute of Husbandry and Pharmaceutical Sciences, Chinese Academy of Agricultural Sciences, Lanzhou, China; ^3^Department of Pediatrics, Ayub Medical College, Abbottabad, Pakistan; ^4^Department of Children Ward, Combined Military Hospital, Abbottabad, Pakistan

## Abstract

**Aim:**

This research study aims to examine the professional quality of life (ProQOL) among healthcare workers (HCWs) in Pakistan during the fourth wave of COVID-19.

**Background:**

Under intense pressure to fight the coronavirus disease 2019 (COVID-19) pandemic, HCWs are more likely to experience psychological problems. Numerous investigations carried out in the past at various points during the pandemic have shown that COVID-19 has had important detrimental effects on HCWs. However, there are many unknowns with regard to ProQOL for HCWs.

**Methods:**

This is a cross-sectional study conducted with Pakistani HCWs who performed their duties during the fourth wave of COVID-19. Data were collected between January 1 and March 31, 2022. A total of 258 HCWs took part in the study evaluating ProQOL. The significance level was <0.05.

**Results:**

Most respondents were males (79.1%), and 20.9% were females. The scores of secondary traumatic stress (STS), burnout (BO), and compassion satisfaction (CS) were 24.03 ± 3.79, 19.18 ± 2.92, and 35.29 ± 4.37, respectively. Compared with higher-income groups, HCWs with lower incomes were significantly (*P* < 0.001) more likely to experience psychological issues. Males had lower BO and STS than female HCWs (*P* < 0.001). Similarly, doctors had a lower STS than nurses (*P* < 0.05). HCWs who worked hours per day longer had a heavier STS (*P* < 0.001).

**Conclusion:**

This study shows low BO levels, moderate CS levels, and STS levels among HCWs. HCWs with lower salary were at a higher risk of mental distress due to the pandemic. HCWs who worked for long hours and had less income had more STS and BO. HCWs who were dissatisfied with their works had poor CS. *Implications for Nursing Management*. It is supposed that these results may help HCW managers to improve job satisfaction and rewards while reducing working hours and workload to improve the ProQOL of HCWs fighting COVID-19. The government should focus on the mental health of HCWs, enhancing their satisfaction and allocating sufficient resources.

## 1. Introduction

The novel coronavirus disease 2019 (COVID-19) pandemic rapidly spread over the world, causing a threat to public health [1]. SARS-CoV-2 mostly affects and causes more damage to the respiratory system [2, 3]. On March 11, 2020, the World Health Organization (WHO) proclaimed COVID-19 to be a pandemic after the first case was discovered in Wuhan, China, in December 2019 [4]. This virus takes 2 to 14 days to incubate, and symptoms typically start to manifest during this time [5]. On December 23, 2022, the disease infected 651.9 million confirmed cases of COVID-19, including 6.65 million deaths, across the world, as reported to the WHO [6]. The pandemic affected both developed and developing nations, with India, Brazil, and United States of America (USA) suffering greatly from its effects in terms of mortality and morbidity [7]. In a little period of time, the total number of confirmed cases in emerging or low-resource nations significantly increased. The world economy, particularly China and surrounding developing countries such as Pakistan, Iran, India, Bangladesh, and Afghanistan, is becoming more at risk from COVID-19 [8, 9]. Pakistan has a low- to medium-level economy and 197 million inhabitants, making it a country with a high population density. In Pakistan, from 3 January 2020 to 13 December 2022, there were 1.57 million confirmed cases of COVID-19 with 30,635 deaths, according to the WHO [10]. COVID-19 is known to cause both physical and psychological problems in HCWs [11]. HCWs, such as physicians, nurses, and paramedics, are fighting the pandemic on the frontlines. As is frequently the case, medical staffs coping with such widespread epidemics are susceptible to psychological stress and changes in mood, which impact their health [12].

Healthcare professionals are involved in a variety of tasks, including isolating patients and preventing and controlling infections [13, 14]. In addition, they were at a greater risk of infection during patient care [15]. Although frontline HCWs primarily focus on preventing transmission and caring for COVID-19 patients, the pandemic's effects on mental health and their consequences cannot be underestimated [16]. Healthcare professionals are more frequently experiencing mental health issues such as anxiety, insomnia [17–21], and burnout syndrome (BS) [22]. Meanwhile, stress was a common problem for nurses fighting COVID-19 [23]. During the COVID-19 epidemic, the mental workload of nurses rose [24]. The physical and mental health of nurses may suffer when they work under conditions that raise their risk of infection, pressure, and workload, when there are not enough resources available, and when they have to carry out more physical and psychological labor [25]. The debate over how the COVID-19 epidemic has affected mental health conditions has uncovered glaring gaps in the information that is currently available on the frequency of depression, anxiety, and sleep disturbance amongst HCWs. Therefore, it is essential to identify the mental health burden and deal with it appropriately [26].

Even before the pandemic, there were high levels of burnout and exhaustion among HCWs in Pakistan. According to a cross-sectional study on burnout among 179 HCWs in emergency rooms, 42.4% of them showed emotional fatigue [27]. A study conducted in 2019 that evaluated 118 surgical residents in Karachi discovered that women had greater emotional exhaustion (49.2%) than men did (50.8%). In addition, married individuals showed higher degrees of personal fulfilment and sleep deprivation than single individuals did [28].

A study conducted in six hospitals in Pakistan using the PHQ-9 and GAD-7 scales to evaluate 400 healthcare workers during the first wave of COVID-19 revealed that 21.8% of them had moderate-to-severe anxiety or depression [29]. Similar results were found in a study conducted on 398 healthcare workers from Punjab, where the prevalence of anxiety and depression was 21.4% and 21.9%, respectively [30]. A study of 87 HCWs during the second COVID-19 wave found that 54% of participants experienced psychological fatigue, 77% experienced depersonalization, and 31% experienced low personal accomplishment. These symptoms were all linked to a history of COVID-19 infection or contact with patients who had such a history [31]. As of January 27, 2021, a third-wave novel SARS-CoV-2 variant from the UK, also known as B.1.1.7, had been identified in over 64 countries, including Pakistan. With an average of 100 individuals dying away in Pakistan every day, this B.1.1.7 variant is linked to a higher risk of death than other variants, implicating more burdens on healthcare professionals [32]. In Pakistan, higher levels of burnout were observed in the COVID-19 fourth wave, likely as a result of the pandemic's cumulative physical and emotional repercussions; however, much is still not entirely clear at this time [33]. It is important to comprehend the long- and short-term effects of CS, BO, and SCS in a lower-middle-income nation such as Pakistan. These effects include decreased healthcare quality, an increase in errors, and a decrease in ProQOL, low job satisfaction, and significant costs [33, 34]. However, during the fourth wave of the pandemic, Pakistan did not routinely report on the prevalence of CS, BO, and STS in HCWs. Therefore, in light of the fourth wave of the COVID-19 outbreak, the purpose of our study is to evaluate the levels of the contextual variables depressive concerns, BO, CS, and STS among HCWs in Pakistan.

## 2. Methods

### 2.1. Study Design and Participants

To learn more about the experiences of frontline HCWs caring for patients with COVID-19, a cross-sectional study was carried out. The study was conducted at two governmental health institutions, namely, Ayub Teaching Hospital and Combine Military Hospital Abbottabad, Pakistan. The inclusion criteria for participation in this study were as follows: (1) voluntary participation in the study; (2) healthcare workers on the frontline against COVID-19; and (3) healthcare workers with direct bedside care experience involving COVID-19 patients. Exclusion criteria were as follows: nonfrontline HWCs involved in scientific research, teaching, and hospital administration [13]. Because there were nurses characterized by different levels of experience at least 5–10 times as many variables were represented in the sample size [13]. The current study included 20 variables. A total of 286 questionnaires were collected, 258 questionnaires were included for the final data analysis after those that did not match the requirements were removed.

### 2.2. Measures and Instruments

The relevant published articles and scientific literature on COVID-19 were examined to create a structured questionnaire [13, 26]. The questionnaire was administered in the English language, which is the official language of Pakistan. Three different sections were made in the questionnaire used: (1) sociodemographic information; (2) healthcare worker work conditions and COVID-19 pandemic-related aspects; and (3) mental health conditions [13]. The demographic features comprised of HCWs are their gender, marital status, age, work experience, educational attainment, the infection status (COVID-19) of friends or family members of the HCW, the number of night shifts worked per week, the number of working hours during the epidemic, job satisfaction, pay satisfaction, and work load [13]. The ProQOL Scale was used to measure ProQOL. The ProQOL Version 5 was the instrument used; it consists of 30 items organized into three subscales: burnout (BO), compassion satisfaction (CS), and secondary traumatic stress (STS). The following items were used to test CS: 3, 6, 12, 16, 18, 20, 22, 24, 27, and 30. Items associated with burnout (items 1, 4, 8, 10, 15, 17, 19, 21, 26, and 29 of the ProQOL tool) and STS (items 2, 5, 7, 9, 11, 13, 14, 23, 25, and 28) were included in order to quantify CF. A 5-point Likert scale was used to record responses to these questions, with 1 indicating never, 2 very rarely, 3 sometimes, 4 often, and 5 indicating very often [35]. In published papers, the ProQOL shows high validity and reliability [36, 37]. According to Cronbach's alpha values of 0.89 for CS, 0.83 for BO, and 0.78 for STS in the primary research study, the ProQOL tool was considered reliable. Therefore, a Cronbach's alpha of 0.80 or higher is regarded as particularly desirable for all aspects [38]. The healthcare worker participants were contacted by WhatsApp, and they were informed about the study's purpose and the researchers' interest in the research topic. The hospital administrators providing respondents who were battling COVID-19 were contacted to discuss the goal of our investigation. Those who were not medical professionals and did not finish the assessment were excluded from this survey.

### 2.3. Data Collection

Data were collected between January 1 and March 31, 2022. Google Forms software was used to generate a survey, and the link to it was distributed via WhatsApp. Since every question was needed, only surveys that were completely filled out were collected, ensuring that there were no missing data. The healthcare worker assisted in distributing the surveys individually and through WhatsApp groups. It took approximately 10–15 min to complete the questionnaire.

The combined military hospital in Abbottabad's Ethical Research Committee gave its approval to the current study (CMH Atd-ETH-78-Paed-23). To participate in our online survey, respondents had to assent (accept or deny) to the informed consent declaration on the first page.

### 2.4. Statistical Analysis

Data collected from respondents were directly deposited in Google Worksheets and later transfer into Microsoft Excel and SPSS 22.0. The data analysis, which was based on the quantitative analysis, combined descriptive and inferential statistics. Descriptive statistics (univariate analysis) were performed for each main variable of the study, whether it was an independent or dependent variable, using frequency counts, percentages, and mean ± standard deviation (SD). The socioeconomic characteristics of the individuals and variables associated with HCWs were examined using the chi-square test. The ProQOL manual states that the responses to items 1, 14, 15, and 17 should be reversed to become (1 = 5) (2 = 4) (3 = 3) (4 = 2) (5 = 1). The ProQOL was at a low level in the end, with sum answers of ≤22 and a moderate level with sum answers of 23–41; the ProQOL was at a high level, and the sum answers were ≥42 [35]. The significance level was <0.05.

## 3. Results

### 3.1. Demographic Characteristics

A total of 258 healthcare workers (204) (79.1%) were males, and 54 workers (20.9%) were females. Regarding marital status, 31.3% were single, and 68.7% were married. Regarding age, 50% were 18–30 years old, 43% were 31–40 years old, and 6.97% were >40 years old. On the basis of education, 32.6% had graduated, and 65.1% had postgraduate or above education. Moreover, 2.3% were senior medical students in the last stage of their undergraduate studies. In this study, 76.7% of the participants were physicians, 10.5% were nurses, 11.6% were technicians, and 1.2% were assistants. Furthermore, we found 5.8% of participants from the infectious disease department, 9.3% from the surgical department, 3.5% from the ICU, 15.1% from internal medicine, 16.3% from emergency, and 50% from other departments. Among all participants, 37.2% were satisfied with their monthly salary, 29% were not satisfied, 6.9% were very satisfied, and 26.7% were normal. Regarding work burden, 44.2% showed a heavy work burden. The time interval of duty shown by the participant included 38.3% working for 8 h per day, 57% working for 9–12 h per day, and 4.6% working for 12 h per day as clinical frontline staff, as shown in Table 1. In addition, 34.9% of HCWs had 2 night shifts per week, 20.9% had 1 night shift per week, and 10.5% had 3 night shifts per week. Here, we found that 61.3% of HCW families were infected with COVID-19, and 68.6% of HCW lives were affected by COVID-19, as shown in Table 1.

### 3.2. Prevalence of ProQOL in Healthcare Workers

The healthcare workers' average scores for CS, BO, and STS were categorized into low, moderate, and high groups. The mean scores ± standard deviation (SD) was as follows: CS was 35.29 ± 4.37, while BO and STS were 19.18 ± 2.92 and 24.03 ± 3.79, respectively. These results indicate that healthcare workers generally experience low levels of STS and moderate levels of CS and BO. Specifically, the results reveal that healthcare workers reported moderate levels of compassion satisfaction (60.5%) to low levels (20.9%), low levels of STS (48.8%) to moderate levels (40.3%), and moderate levels of burnout (48%) to low levels (39.5%). Details are shown in Table 2.

### 3.3. Factors Related to HCWs' ProQOL

In univariate analysis, general data were used as independent factors and anxiety, depression, BO, STS, and CS were used as dependent variables, and the findings demonstrated that HCW age, marital status, number of working hours per day, workload, and job satisfaction were associated with STS; workload, job satisfaction, and salary satisfaction were associated with BO and CS (*P* < 0.05; Table 3). Chi-square analysis was performed, and it showed that males possessed lower anxiety, depression, STS, and compassion satisfaction than female HCWs (*P* < 0.001). Single HCWs had a lower burnout risk than married HCWs (*P* < 0.006). HCWs aged 18–30 had heavier STS than HCWs aged 31–40 and ≥41 (*P* < 0.001). Physicians had lower anxiety and depression, STS, and compassion satisfaction than nurses, technicians, and assistants (*P* < 0.05). The HCWs who worked in the emergency department, ICU, and internal medicine department had higher levels of burnout, anxiety, and depression than those who worked in other departments or units (*P* < 0.05). Those with 1–5 years of work experience had a larger percentage of moderate STS than those with more than 5 years of work experience (*χ*^2^ = 13.738, *P*=0.008). Working hours per day (h) longer, the STS was heavier (*χ*^2^ = 46.748, *P* < 0.001). When the job was more satisfied, the HCWs had lower anxiety and depression (*χ*^2^ = 15.020, *P*=0.005) and secondary traumatic stress (*χ*^2^ = 9.526, *P*=0.009). In addition, the HCWs had more satisfaction by monthly salary, and they had poor levels of burnout, anxiety, depression, STS, and CS (*P* < 0.001) (Table 3).

## 4. Discussion

The current findings demonstrated that during the COVID-19 pandemic, HCWs had a high frequency of self-reported sociodemographic and psychological issues. The prevalence of anxiety, depression, burnout, and general psychological issues varied significantly among different types of healthcare employees. This research revealed several sociodemographic factors associated to HCWs to help officials and policymakers establish policies and improve HCW facilities. Due to their frequent use of empathy, extended exposure to secondary trauma, and work environment, healthcare practitioners are likely to feel depression, anxiety, burnout, and secondary traumatic stress [39]. In addition, the constrained resources and numerous healthcare needs during this time may raise the possibility of compassion fatigue. This evidence has increased the risk of psychological distress due to the pandemic.

According to our research, HCWs had to moderate degrees of anxiety and depression, whereas high levels of depression signs and symptoms were found in Indian healthcare professionals who were classified as having moderate-to-severe depression symptoms (22% of them) [40]. During the COVID-19 pandemic, HCWs who scored higher on fatigue, melancholy, anxiety, and stress also had a greater fear of contracting coronavirus [41]. In China, healthcare professionals and nurses experienced symptoms of despair, anxiety, insomnia, and discomfort two months after the first identified COVID-19 case [42]. Managing COVID-19 patients led to increased anxiety and reduced health-related quality of life [43]. Frontline healthcare workers with moderate-to-severe depression and anxiety symptoms saw a decline in their overall quality of life [40].

According to our research, medical staffs that have been exposed to the COVID-19 pandemic might experience both positive and negative psychological consequences. According to the results, STS and burnout were frequent, although levels of CF varied from moderate to high. While greater levels of CS and less CF were detected in HCWs who reported feeling highly content with their salaries, less BO was seen in HCWs who reported feeling highly satisfied with their earnings (*P* < 0.05). Similarly, during the pandemic, 72.5% of nurses were satisfied with their current job, while 73.6% of nurses were satisfied with their pay [44]. Less BO was experienced by nurses who were satisfied with their pay, while higher CS and less CF were seen in nurses who were highly satisfied with their jobs [13]. During the COVID-19 epidemic, similar findings were reported in Ecuador, where medical staff experienced mild to moderate burnout and CF [45]. Similar findings were made by Jordan et al. in their study, which revealed that high-income nurses had a low incidence of CF [46]. According to Tian et al., nurses who were dissatisfied with their salaries had higher BO [47]. In addition, in the study reported by Niu et al., nurses who reported having low job satisfaction were more likely to suffer from CF, which is consistent with our findings regarding job satisfaction [13].

The results of this study showed that STS scores were greater than the crucial value, as well as higher than those of Turkish nurses prior to the pandemic and Italian nurses during the pandemic [48]. The signs and symptoms of STS can include anxiety, sleeplessness, and intrusive thoughts [49]. Patients with COVID-19 are more likely to experience mood swings, irritability, and grief [13]. HCWs who interact with these patients could feel a great deal of psychological pressure. It has been discovered that nurses fighting the pandemic are more likely to have symptoms of somatization [50]. As a result, it is essential to care about HCWs and STS, offer social and psychological support, promote a healthy team culture, and engage in proper exercise to lessen the pandemic's negative effects on HCWs.

In this study, HCWs with excessive workloads exhibited higher levels of BO and STS (*P* < 0.001), as well as substantially lower CS levels. According to certain research and our study, nurses who work a lot are more likely to experience STS, which lowers job satisfaction and BO [51]. In a 2020 study with 506 Spanish healthcare professionals, 94% showed moderate-to-severe compassion fatigue, 84% showed moderate-to-severe burnout, and 84.4% of the entire sample showed moderate-to-severe compassion satisfaction [52]. Burnout and secondary traumatic stress were more severe in physicians than in nurses. In addition, nurses had higher levels of compassion satisfaction. Another Spanish study of 973 healthcare professionals revealed that 90.6% had high levels of compassion satisfaction, noting that this trait is more common in people between the ages of 35 and 55 and that nurses outnumbered physicians even though nurses were more prevalent [53].

The study's findings showed that the COVID-19 pandemic significantly increased the burden of psychological issues among various healthcare professionals. The results imply that exposure to negative information about the pandemic may be linked to a higher risk of psychological issues. Participating in frontline work appears to be a significant risk factor for anxiety, sleeplessness, and psychological issues in general [54–56]. These results suggest the implementation of measures that go beyond saving patient lives with COVID-19 psychosocial interventions and support for healthcare workers, which will help us better understand the impact of pandemics on psychological health among HCWs. Early in the pandemic, support for transient psychological issues, including anxiety and depression, is required, along with evidence-based psychosocial therapies. Furthermore, stress should be placed on self-relaxation training, regular exercise, and a healthy lifestyle.

### 4.1. Limitations of the Work

This study has certain limitations, even if it adds empirically to the conversation about the ProQOL of HCWs. First, because the study was performed online, self-selection bias could have been a factor. Considering that a convenience sample was used, it is not possible to extrapolate the findings to other situations because they may not accurately represent the population. This study was cross-sectional in design and evaluated ProQOL at a single moment in time within the particular era and COVID-19 pandemic scenario, care must be taken when interpreting the data. As the number of COVID-19 cases continued to rise in Pakistan during the fourth wave, hospitals faced increasing strain from the influx of patients. Government policies proved ineffective due to public indifference. Notably, the private sector hospitals in Pakistan outnumber government-run facilities, and they have a larger contingent of healthcare professionals, particularly physicians and doctors, compared to nurses and other healthcare providers. Consequently, during the fight against COVID-19, physicians had to take on the frontline roles compared to nurses and other professionals following suit [57]. In addition, there is a significant gender disparity in the healthcare workforce, with a higher proportion of men compared to women, largely attributed to the country's ongoing struggle for women's empowerment and education. This gender imbalance is especially prominent in roles such as nursing and nursing assistants, where men dominate the positions [58]. Another limitation of the study is its cross-sectional design, as the pandemic has since subsided and the study cannot capture the long-term impact on the mental health of these frontline healthcare workers. To address this, it would be advisable to conduct a longitudinal study to track the evolution of the mental health symptoms assessed in this research over time.

## 5. Implications for Nursing Management

Prior to fighting the epidemic, it is essential to teach HCWs about patient knowledge, epidemic control and preventive techniques and to build personal resiliency. Programs for stress management and peer social support are also essential to lower BO and STS levels, and they should offer efficient interventions to increase HCWs' satisfaction levels. The foundation of the healthcare system's physical and mental health must be protected; thus, governments must take the necessary action. Financial subsidies and rewards from the government are advised to increase work satisfaction for HCWs. According to our research, structural empowerment techniques can be developed by hospital administration and HCWs to improve HCWs' psychological empowerment. We also advocate for the development of counseling services, assistance, and programs to support HCWs' psychological well-being. These measures will help preserve a strong team culture.

## 6. Conclusion

The HCWs have moderate levels of compassion satisfaction, secondary traumatic stress, and low levels of burnout. High levels of perceived susceptibility and severity, as well as elevated psychological distress, are present in HCWs battling COVID-19. Healthcare professionals' levels of anxiety and CF may be impacted by professional contextual factors (poor income and lengthy hours). HCWs with a heavy workload and little pay had more severe depression and STS. The administration of the hospital must to keep a low degree of burnout, increase CS levels, manage STS levels, and provide HCWs with a good work environment. Further research studies with different research approaches and different low-resource sceneries are suggested to address the professional quality of life level.

## Figures and Tables

**Table 1:** Sociodemographic and work-related characteristics of 258 frontline HCWs against COVID-19.

Variable	*N* (%)
Gender	
Male	204 (79.1)
Female	54 (20.9)
Marital status	
Single	81 (31.4)
Married	177 (68.6)
Age	
18–30	129 (50.0)
31–40	111 (43.0)
>41	18 (7.0)
Education	
Graduate	84 (32.6)
Postgraduate	168 (65.1)
Junior	6 (2.3)
Professional title	
Physicians	198 (76.7)
Nurse	27 (10.5)
Technician	30 (11.6)
Assistant	3 (1.2)
Monthly salary	
<50,000 PKR	54 (20.9)
50,000–100,000 PKR	132 (51.1)
>100,000 PKR	72 (28.0)
Workload is very heavy	
Yes	114 (44.2)
No	24 (9.3)
Normal	120 (46.5)
Number of night shifts per week	
0	87 (33.7)
1	54 (20.9)
2	90 (34.9)
≥3	27 (10.5)
Family infected with COVID-19	
Yes	159 (61.6)
No	99 (38.4)
COVID-19 has affected eating habits	
Yes	120 (46.5)
No	123 (47.7)
Normal	15 (5.8)
COVID-19 has affected my job	
Yes	171 (66.3)
No	87 (33.7)
Satisfaction by monthly salary	
Very satisfied	18 (7.0)
Satisfied	96 (37.0)
General	69 (27.0)
Not satisfied	75 (29.0)
Work experience (years)	
1–5	138 (53.5)
5–10	84 (32.5)
>11	36 (14.0)
Working hours per day (h)	
<8	99 (38.4)
9–12	147 (57.0)
>12	12 (4.6)
Department	
ICU	9 (3.5)
Infectious disease ward	15 (5.8)
Surgical	24 (9.3)
Internal medicine	39 (15.1)
Emergency dept	42 (16.3)
Other	129 (50)
Job satisfaction	
Very satisfied	42 (16.3)
Satisfaction	135 (52.3)
General	60 (23.3)
Not satisfied	21 (8.1)
COVID-19 has affected personal life	
Yes	177 (68.6)
No	60 (23.3)
Normal	21 (8.1)
COVID-19 has affected sleeping pattern	
Yes	117 (45.3)
No	123 (48.0)
Normal	18 (7.0)
Are you worried about COVID-19?	
Yes	156 (60.5)
No	78 (30.2)
Normal	24 (9.3)
Have accomplished all job tasks?	
Yes	189 (73.2)
No	42 (16.3)
Rare	27 (10.5)

**Table 2:** Prevalence of ProQOL in healthcare workers (*n* = 258).

Variables	Categories	*n*	Prevalence (%)
Burnout	Yes	44	17

Secondary traumatic stress	Yes	144	56.2

Compassion satisfaction	Yes	124	48

Burnout	Low	102	39.5
	Moderate	123	48
	High	12	12.5

Anxiety and depression	Low	111	43
	Moderate	120	46.5
	High	27	10.5

Secondary traumatic stress	Low	126	48.8
	Moderate	102	40.3
	High	30	10.9

Compassion satisfaction	High	48	18.6
	Moderate	156	60.5
	Low	54	20.9

**Table 3:** Factors related to HCWs' professional quality of life.

Variable	Burnout			Anxiety and depression			Secondary traumatic stress			Compassion satisfaction		
	Low	Moderate	High	Low	Moderate	High	Low	Moderate	High	Low	Moderate	High
Gender												
Male	87	90	27	99	90	15	114	72	18	33	132	39
Female	15	33	6	12	30	12	14	28	12	21	24	9
*χ*^2^	5.125			17.097			17.335			13.089		
*P*	0.077			0.001			0.001			0.001		
Marital status												
Single	39	27	15	31	35	15	35	34	12	15	48	18
Married	63	96	18	84	75	18	99	60	18	39	102	36
*χ*^2^	10.337			4.090			3.758			0.448		
*P*	0.006			0.129			0.153			0.799		
Age												
18–30	45	60	24	34	69	26	41	39	31	36	56	19
31–40	29	63	19	37	67	25	42	63	6			
≥41	4	9	5	3	10	5	7	9	2	3	11	4
*χ*^2^	4.014			1.529			24.626			3.346		
*P*	0.404			0.820			0.001			0.188		
Professional title												
Doctor	75	102	21	87	87	24	69	112	18	66	123	9
Nurse	9	13	5	7	17	4	4	21	2	2	25	0
Technician	8	17	5	3	25	2	5	22	3	7	17	3
Assistant	1	2	0	0	3	0	0	3	0	1	2	0
*χ*^2^	2.691			23.597			9.964			20.096		
*P*	0.611			0.001			0.041			0.001		
Department/unit												
Other	59	57	13	54	66	9	42	81	6	33	89	7
Emergency	15	17	10	7	22	13	13	25	4	0	0	0
ICU	2	3	4	1	4	4	4	3	2	5	3	1
Internal medicine	10	18	11	8	23	9	7	27	5	6	31	2
*χ*^2^	14.387			25.929			5.534			0.160		
*P*	0.006			0.001			0.237			0.923		
Work experience (years)												
1–5	54	66	18	49	74	15	32	102	4	17	113	9
5–10	32	46	6	18	56	10	11	67	7	15	61	8
11	9	23	3	7	25	2	14	19	3	3	29	2
*χ*^2^	5.231			8.146			13.738			3.093		
*P*	0.264			0.086			0.008			0.542		
Working hours per day (h)												
8	34	56	9	20	71	8	45	48	6	23	67	9
9–12	39	84	24	37	99	11	23	117	7	34	106	7
12	4	5	3	3	5	4	9	3	0	3	9	0
*χ*^2^	5.096			4.922			46.748			3.404		
*P*	0.278			0.295			0.001			0.493		
Job satisfaction												
Very satisfied	4	38	0	4	36	2	2	40	0	3	38	1
Satisfied	23	94	12	31	89	9	15	12	2	21	104	4
General	15	42	3	5	47	8	3	56	1	4	54	2
Not satisfied	2	15	4	3	7	11	5	7	9	4	11	6
*χ*^2^	5.931			15.020			9.526			5.828		
*P*	0.204			0.005			0.009			0.054		
Satisfaction by monthly salary												
Very satisfied	3	15	0	2	13	3	6	12	0	9	9	0
Satisfied	12	75	9	23	66	7	13	80	3	22	72	2
General	9	52	8	14	48	13	11	60	4	0	53	0
Not satisfied	5	38	32	20	38	35	13	44	12	17	38	14
*χ*^2^	22.660			29.122			58.711			29.096		
*P*	0.001			0.001			0.001			0.001		

## Data Availability

The data used to support the findings of this study are available from the corresponding author upon request. The data are not publicly available because of privacy or ethical restrictions.
